# Multimodal Particulate Matter Prediction: Enabling Scalable and High-Precision Air Quality Monitoring Using Mobile Devices and Deep Learning Models

**DOI:** 10.3390/s25134053

**Published:** 2025-06-29

**Authors:** Hirokazu Madokoro, Stephanie Nix

**Affiliations:** Faculty of Software and Information Science, Iwate Prefectural University, Takizawa 020-0693, Iwate, Japan; nix_s@iwate-pu.ac.jp

**Keywords:** Contrastive Language–Image Pre-Training, particulate matter, transformer backbone, single-board computers, mobile cameras

## Abstract

This paper presents a novel approach for predicting Particulate Matter (PM) concentrations using mobile camera devices. In response to persistent air pollution challenges across Japan, we developed a system that utilizes cutting-edge transformer-based deep learning architectures to estimate PM values from imagery captured by smartphone cameras. Our approach employs Contrastive Language–Image Pre-Training (CLIP) as a multimodal framework to extract visual features associated with PM concentration from environmental scenes. We first developed a baseline through comparative analysis of time-series models for 1D PM signal prediction, finding that linear models, particularly NLinear, outperformed complex transformer architectures for short-term forecasting tasks. Building on these insights, we implemented a CLIP-based system for 2D image analysis that achieved a Top-1 accuracy of 0.24 and a Top-5 accuracy of 0.52 when tested on diverse smartphone-captured images. The performance evaluations on Graphics Processing Unit (GPU) and Single-Board Computer (SBC) platforms highlight a viable path toward edge deployment. Processing times of 0.29 s per image on the GPU versus 2.68 s on the SBC demonstrate the potential for scalable, real-time environmental monitoring. We consider that this research connects high-performance computing with energy-efficient hardware solutions, creating a practical framework for distributed environmental monitoring that reduces reliance on costly centralized monitoring systems. Our findings indicate that transformer-based multimodal models present a promising approach for mobile sensing applications, with opportunities for further improvement through seasonal data expansion and architectural refinements.

## 1. Introduction

Despite the declining trend in Particulate Matter (PM) emissions due to various environmental measures, significant concerns about their health impacts persist. PM primarily originates from human activities such as vehicle exhaust emissions, industrial processes, construction operations, and the combustion of wood and coal [1,2]. Much of this PM is formed through highly complex atmospheric reactions involving sulfur oxides, nitrogen oxides, and volatile organic compounds [3]. These particles are believed to enter the body via the respiratory system, adversely affecting the lungs and vascular system. With the proliferation of affordable, compact, and lightweight sensors, it has become feasible to measure various particulate matter concentrations in both indoor and outdoor environments [4].

However, predicting PM concentrations remains extremely challenging [5] due to the multitude of factors influencing changes, including meteorological and geographical characteristics, as well as the increasing complexity of emission sources. Numerous aspects of this phenomenon remain unexplained. Current air pollution prediction models [6] allow for qualitative assessments, such as rough increases or decreases in concentration and short-term forecasts of up to 1 h ahead. However, challenges persist in achieving accurate quantitative predictions of PM concentrations and long-term forecasts. One contributing factor was the historical reliance on the Long Short-Term Memory (LSTM) model [7], introduced in 1997, prior to the emergence of the transformer architecture [8], unveiled in 2017.

While the backbone of convolutional neural networks (CNNs) was proposed over 30 years ago [9], the development of multilayered architectures and the subsequent deep learning boom began a decade ago [10]. Since then, CNN-based models have achieved significant performance improvements [11]. In recent years, transformer backbones [8] have gained substantial attention, marking a progressive shift from CNNs to transformer architectures [12]. Transformer architectures now outperform CNN-based models [13] in terms of performance, although challenges persist regarding computational demands and the requirement for large-scale datasets during pre-training. Moreover, relatively few studies have focused on estimating PM concentration distributions from images, despite the extensive research advancements and practical implementations observed in image classification and segmentation since the deep learning boom began [14].

**Figure 1:**
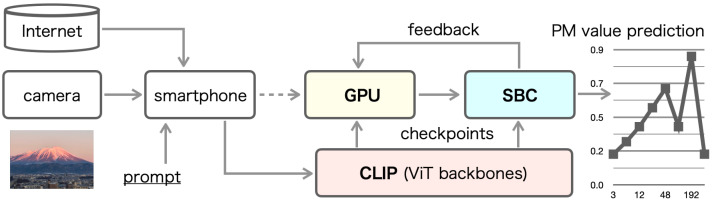
Overall structure of our proposed system prototype.

This study aims to develop a framework for a novel application task for air quality measurement by leveraging transformer-based architectures. To achieve this, we implemented a specialized system incorporating accelerators that facilitate efficient and reliable execution of transformer models. Figure 1 illustrates our proposed system prototype, which integrates the necessary components to acquire input images and predict PM concentration. A smartphone-integrated camera was designed to capture real-time environmental data, serving as the primary input for image acquisition. This study proposes an automatic method for generating pre-training datasets using vision-based and language-based embedding models, offering a novel application of deep learning in environmental monitoring.

## 2. Related Work

Numerous studies have been conducted to predict particulate matter with an aerodynamic diameter of 2.5 μm or less (PM_2.5_) using deep learning models, primarily Long Short-Term Memory (LSTM)-based methods [7,15,16,17,18,19,20,21,22,23,24,25,26,27,28,29,30,31,32,33,34,35,36,37,38,39,40,41,42,43,44], including our previous work [45]. Table 1 summarizes five representative studies related to our research. These existing studies focus on enhancing PM forecasting through the integration of advanced machine learning techniques and diverse data sources across different regions, such as Seoul, South Korea, and Los Angeles County, California. Koo et al. [46] developed a hybrid ConvLSTM-DNN model that effectively captures spatiotemporal dependencies, outperforming traditional models like Community Multiscale Air Quality (CMAQ), particularly during peak pollution periods. Similarly, Feng et al. [47] emphasized the importance of combining ground sensor data, meteorological features, and machine learning to improve air quality forecasting, highlighting the resilience of their hybrid models against forecast inaccuracies. Jianyao et al. [48] expanded the approach to China, integrating various datasets to enhance understanding of PM_2.5_ distribution and improve short-term forecasting accuracy. Zhang et al. [49] introduced a novel method using drone-captured images to estimate PM concentrations, demonstrating a cost-effective alternative to traditional monitoring stations. Dai et al. [50] introduced a novel LUR-GBM model that integrates land-use regression, the Kriging method, and LightGBM to estimate PM_2.5_ concentrations across China, achieving high prediction accuracy and providing insights into the spatial and temporal dynamics of PM_2.5_ pollution. Collectively, these studies underscore the critical role of advanced modeling techniques and diverse data integration in addressing air quality challenges and supporting public health initiatives.

Koo et al. [46] focused on improving the accuracy of PM_2.5_ forecasts in Seoul, South Korea, by combining machine learning techniques with traditional models such as CMAQ and Weather Research and Forecasting (WRF) systems. They proposed a hybrid method that integrates ConvLSTM networks with Deep Neural Networks (DNNs) to predict 6 h average PM_2.5_ concentrations up to 72 h ahead, using 12 time steps in the forecasting process. They used air quality datasets obtained from the AirKorea website, which sources PM_2.5_ data from 1196 monitoring stations in China and 416 monitoring stations in South Korea. The proposed ConvLSTM-DNN model was particularly noted for its robustness in handling medium-range forecast horizons, where it maintained high predictive accuracy by effectively capturing both spatial and temporal dependencies of PM_2.5_ concentration distributions. Although the hybrid model’s superior performance was attributed to its ability to combine the strengths of ConvLSTM for spatiotemporal forecasting and DNNs for non-linear feature extraction, transformer-based backbones exhibited superior performance in reducing forecast inaccuracies as the prediction horizon increased [54]. However, their study did not conduct comparative experiments with state-of-the-art deep learning architectures.

Feng et al. [47] proposed a hybrid forecasting framework for PM_2.5_ concentrations by integrating the CMAQ atmospheric chemistry transport model with LSTM and random forest (RF), which are widely used in traditional machine learning approaches. The system provided 1 km × 1 km spatial resolution with hourly forecasts. The LSTM component achieved significantly improved accuracy over CMAQ alone: an RMSE of 3.66 μg/m^3^ for 1 h forecasts versus CMAQ’s 45.81 μg/m^3^. Wavelet transform enabled the LSTM to capture PM_2.5_ variations across multiple time scales, enhancing its temporal resolution. The RF model leveraged LSTM outputs combined with meteorological and topographical data, where CMAQ results served as a key input feature. This hybrid approach achieved at least 42.3% improved forecasting accuracy compared to CMAQ alone, demonstrating strong potential for predicting other air pollutants. Such performance supports informed decision-making in pollution management and health risk reduction.

Jianyao et al. [48] focused on enhancing PM_2.5_ forecasting and understanding its spatial and temporal distribution in China, leveraging diverse data sources and advanced machine learning techniques. Their study integrated ground monitoring data from the China Geographic Monitoring Cloud platform, satellite imagery from NASA’s MAIAC and MODIS missions, and meteorological data from the European Centre for Medium-Range Weather Forecasts (ECMWF) via ERA5. Their research employed a hybrid machine learning model combining ConvLSTM with deep neural networks (DNNs) to forecast PM_2.5_ levels up to 72 h into the future. Their model used historical input features from meteorological data, air quality observations, and CMAQ forecasts to make predictions. Their approach addresses the limitations of traditional models like CMAQ, which can overestimate or underestimate PM_2.5_ concentrations during peak pollution periods. By providing robust and accurate forecasts of PM_2.5_ concentrations, their research aims to support better decision making for air quality management and public health crisis response.

Zhang et al. [49] proposed a novel method for estimating PM_2.5_ concentrations using image data to address the limitations of traditional air monitoring stations, which are expensive and sparsely distributed. The authors developed PMEstimatingNet, a deep neural network that analyzes haze-relevant features extracted from drone-captured images to predict air quality with high spatial resolution. Their approach extracted six key haze-related visual features: refined dark channel, max local contrast, max local saturation, min local color attenuation, hue disparity, and chroma. These features were processed through a CNN architecture that effectively correlated visual information with particulate matter levels. The researchers also collected a comprehensive dataset containing both high-resolution PM_2.5_ measurements from an array of nine ground sensors and corresponding multiview drone imagery. Their experimental results demonstrated impressive performance, significantly outperforming single-view approaches.

Dai et al. [50] introduced a novel LUR-GBM model that integrates land-use regression, the Kriging method, and LightGBM to estimate PM_2.5_ concentrations across China from 2016 to 2021. The model utilizes data from monitoring stations, incorporating various factors such as land use, meteorology, topography, vegetation indices, population density, traffic, and pollution sources to accurately predict PM_2.5_ levels. The model’s performance was rigorously evaluated using a ten-fold cross-validation approach, demonstrating superior prediction accuracy compared to other machine learning models, including BPNN, DNN, RF, XGBoost, and LightGBM. Their findings revealed a distinct spatial distribution of PM_2.5_ concentrations characterized by higher concentrations in the eastern regions and lower levels in the west influenced significantly by topographical features, alongside notable seasonal variations.

Mohan et al. [51] proposed En3C-AQI-Net, an innovative ensemble model designed for accurate air quality estimation in Delhi, India. Their approach leverages cutting-edge technologies by combining three distinct deep learning models: a fine-tuned Data-Efficient Image Transformer (DeiT) for processing outdoor images, a specialized CNN incorporating a dark-channel prior for feature extraction, and a one-dimensional CNN trained on meteorological parameters. Their proposed model employed weighted average ensemble learning to synthesize predictions from these three components, enabling both classification into six AQI categories and precise numerical AQI estimation. The experimental results demonstrated the considerable superiority of En3C-AQI-Net over pre-trained CNN models. The En3C-AQI-Net architecture successfully captured diverse and complementary features from heterogeneous data sources, proving particularly effective in Delhi’s challenging context characterized by extreme seasonal variations in pollution levels.

Ahmed et al. [52] introduced AQE-Net, a deep learning model designed to estimate air quality indices from mobile camera images, addressing the need for efficient and inexpensive air quality monitoring tools. Their proposed model was developed and evaluated using the KARACHI-AQI dataset, which consists of 1001 hourly samples collected from an air quality monitoring station in Karachi, Pakistan, over a three-month period from August to October 2021, with each sample containing photographs, PM_2.5_ measurements, and corresponding AQI values. The architecture of AQE-Net incorporated Spatial and Context Attention (SCA) blocks integrated with a ResNet18 backbone, enabling continuous adjustment of feature relevance through self-supervision modules that analyzed spatial relationships and contextual information within the images. Experimental results demonstrated that AQE-Net significantly outperformed traditional machine learning approaches and older deep learning architectures.

As the most recent approach, Ahmed et al. [53] introduced Air Quality Prediction-Mamba (AQP-Mamba), a sophisticated video-based deep learning architecture that constitutes a major leap forward in air quality monitoring technology. Unlike previous approaches that relied on static images or outdated neural network architectures, AQP-Mamba leverages the cutting-edge Structured Selective State Space Model (SSSM) [55] with a selective scan mechanism coupled with a hybrid predictor to effectively process and analyze temporal variations in air pollution. Their design allowed AQP-Mamba to simultaneously perform multiple tasks, including regression for specific pollutant concentrations of PM_2.5_ and PM_10_, alongside classification of AQI categories, making it exceptionally versatile for environmental monitoring applications. By effectively addressing the limitations of previous research that relied on outdated architectures like ResNet18 or even transformer-based models with quadratic complexity, AQP-Mamba establishes a new benchmark for air quality estimation systems that is not only more accurate but also more computationally efficient and scalable, potentially revolutionizing environmental monitoring in regions where traditional instrumentation is prohibitively expensive or impractical to deploy.

## 3. PM Prediction from 1D Time-Series Signals

Prior to exploring PM value prediction based on 2D image data, we conducted preliminary experiments to investigate the feasibility of predicting PM concentrations from one-dimensional signal waveforms. For this task, we evaluated several machine learning models designed for handling temporal data, including LSTM [7] as a modified version of Recurrent Neural Networks (RNNs), transformers [8] and their derivatives, as well as linear models [56]. These approaches were chosen to analyze patterns in time-series signals and assess their potential for capturing dynamic changes in PM concentrations. By comparing their performance on one-dimensional data, we aimed to lay the groundwork for future research that integrates both image-based spatial and signal-based temporal modalities, enabling more comprehensive environmental monitoring solutions. We also employ LSTM to predict vertical PM distribution using a drone [45].

RNNs are a type of neural network designed for processing time-series or sequential data. A key advantage is their ability to retain information from previous inputs, enabling them to influence subsequent outputs. However, RNNs suffer from the gradient vanishing problem, which hinders their capacity to learn long-term dependencies in sequences. Elman-type networks [57] are a variant of RNNs with a relatively simple structure. They incorporate a hidden layer that retains information from previous time steps by passing the hidden state to the next time step. Essentially, the network consists of three layers: an input layer, a hidden layer, and an output layer. The output of the hidden layer is fed into the next time step. Elman networks learn through backpropagation through time (BPTT) [58], which propagates errors through the sequence to adjust weights effectively, enabling the model to capture temporal dependencies in sequential data.

LSTM [7] networks are an advanced variant of RNNs designed to address the gradient vanishing problem and improve the ability to learn long-term dependencies in sequential data. At their core, LSTM networks incorporate a memory cell that retains information over extended time intervals. To regulate the flow of information, they employ three types of gates: forget gates, input gates, and output gates. These gates control whether information is retained, updated, or outputted at each time step, enabling the network to selectively process and store relevant data. Forget gates determine which information to discard from the memory cell. Input gates regulate new information entering the memory cell. Output gates control the output of information from the memory cell.

This mechanism allows LSTMs to selectively retain relevant information and discard unnecessary details, enabling them to model complex temporal patterns effectively. Gated Recurrent Units (GRUs) [59] are a simplified version of LSTMs designed for computational efficiency. Unlike LSTMs, GRUs do not include a separate memory cell but instead use a update gate that combines the functions of the input and forget gates. This streamlined architecture reduces computational load and speeds up training. However, while GRUs are efficient for many tasks, they may underperform compared to LSTMs in specific scenarios requiring precise control over long-term dependencies.

Zeng et al. [56] evaluated advanced architectures such as the transformer [8], Informer [60], Autoformer [61], FEDformer [62], and Pyraformer [63], highlighting each model’s ability to capture complex dependencies in time series data. These models leverage sophisticated mechanisms like self-attention in transformers to effectively process long-range contextual information, enabling more accurate and robust performance in sequential prediction tasks. The comparative analysis between different models highlights their suitability for specific tasks and datasets, providing guidance for practical applications. However, these models often require substantial computational resources due to their complexity, resulting in high training time and memory usage. For example, architectures like transformers or FEDformer may pose challenges to scalability and deployment. Simpler models lack the capacity to capture intricate patterns, limiting their effectiveness on complex tasks. Additionally, overfitting and performance degradation under data scarcity remain concerns, necessitating careful hyperparameter tuning and data augmentation for optimal results.

The transformer [8] model differs from traditional deep learning architectures by eliminating recurrent layers and convolutional layers, relying solely on attention mechanisms for learning. During training, the attention layer assigns weights to input data, outputting vector quantities that reflect contextual importance based on relevance. While parallel processing enables faster learning compared to LSTM models, transformers typically require more memory due to their architecture. Variants of the transformer include Informer [60], Autoformer [61], Pyraformer [63], and FEDformer [62]. Informer [60] enhances computational efficiency by incorporating ProbSparse self-attention to reduce computation while maintaining performance. Autoformer [61] distinguishes itself through its ability to decompose trends step by step during prediction processes. Pyraformer [63] improves modeling of multiscale temporal relationships by combining both scale-intra attention (within-resolution dependencies) and scale-inter attention (across-resolution dependencies). FEDformer [62] integrates Fourier transforms and wavelet transforms to apply attention operations in the frequency domain, enhancing its suitability for long-term forecasting tasks through specialized handling of frequency-based patterns.

### 3.1. Time-Series Datasets

The Japanese archipelago, situated at the eastern edge of the Eurasian continent, is an arc-shaped chain of islands located along the boundary between the Asian continent and the Pacific Ocean. The Japanese islands are positioned in the mid-latitude region and are significantly influenced by the westerly winds. These winds, driven primarily by the temperature contrast between the polar regions and the tropics, play a key role in shaping Japan’s climate and weather patterns. As a result of Japan’s involvement in the Asia-Pacific War, which was part of World War II, Japan experienced severe air pollution primarily composed of PM during its great period of rapid economic growth. This pollution was largely driven by industrialization and urbanization. However, the implementation of environmental regulations such as the Air Pollution Control Act and rising public awareness have led to a significant decline in average PM concentrations over recent decades. While Japan’s economic slowdown has contributed to this reduction, China’s rapid economic development has instead fueled severe air pollution on its mainland, highlighting the complex interplay between industrialization, policy, and environmental outcomes across Asia [64].

The transboundary transport of these pollutants to the Japanese archipelago has resulted in sudden increases in concentration levels, raising concerns about adverse health effects including respiratory and cardiovascular diseases [65]. Particularly in western Japan, there are days when PM measurements exceed environmental standards across wide areas. In the Tohoku region as well, PM transported over the Sea of Japan by westerly winds can be observed at high concentrations across extensive areas, as there are no mountain ranges to block their passage. Furthermore, in rice cultivation areas that support the staple food of the Japanese population, the burning of rice straw—practiced for crop residue disposal and pest control—persists despite advancing regulations through local ordinances. This practice continues to be problematic as a localized source of particulate matter, including PM_2.5_ (particles with a diameter of 2.5 μm or less) [65].

This research aims to develop a system that predicts the temporal PM distribution using only cameras mounted on mobile devices, leveraging cutting-edge deep learning technology. The large-scale distribution of PM is currently monitored extensively and in real time by measurement stations. These stations are primarily installed at roadside locations and operated by the Ministry of the Environment across over 1000 sites throughout Japan. Monitoring data can be accessed in real time via the official website “Soramame-kun.” Meanwhile, recent advances in sensor technology have enabled widespread use of affordable, compact, and lightweight sensors [66], facilitating straightforward measurement of PM levels in both indoor and outdoor environments. Song et al. [67] developed a deep-learning-based method for predicting localized PM distribution by utilizing visual data from smartphone-captured images, without using optical scattering sensors. However, the backbone network serving as the core of their deep learning approach is outdated, creating opportunities for performance optimization. Moreover, challenges persist in time-series prediction for analyzing long-term trends and in integrating multisensor measurements to produce two- or three-dimensional forecasts.

Based on our previous research [68], we collected PM_2.5_ concentration measurements on the rooftop at the Graduate School Building on the Akita Campus of Akita Prefectural University (latitude: $39^{\circ }48^{\prime }11^{\prime \prime }$ N, longitude: $140^{\circ }2^{\prime }46^{\prime \prime }$ E) from 23 May to 12 October 2022. The raw data, recorded at 2 s intervals, were converted into hourly intervals using a moving average. The dataset was divided into training, validation, and test subsets with a ratio of 7:1:2. A Look-Back (LB) window *T* was set to {3,6,12,24,48,96,192,336} time steps. The monitored parameters include PM_2.5_ ($\mu g/m^{3}$), PM_10_ ($\mu g/m^{3}$), temperature (°C), humidity (%), and atmospheric pressure (hPa).

### 3.2. Evaluation Metrics

In machine learning studies, the Mean Squared Error (MSE) and Mean Absolute Error (MAE) are two widely used metrics for assessing the performance of regression models [69]. While both measures evaluate the discrepancy between predicted values and actual observations, they differ in their mathematical formulations, leading to distinct statistical characteristics. The MSE penalizes larger errors more heavily due to its squared term, whereas the MAE provides a more intuitive interpretation of the average error magnitude.

The MSE quantifies the average of the squares of the errors, where errors are defined as the differences between predicted and actual values. The mathematical formulation of MSE is expressed as

(1) $$\mathrm{MSE}=\frac{1}{n}\sum_{i=1}^{n}{\left(y_{i}-{\hat{y}}_{i}\right)}^{2},$$

where *n* represents the number of observations, $y_{i}$ denotes the actual value, and ${\hat{y}}_{i}$ indicates the predicted value for the *i*-th observation.

The MSE exhibits several notable characteristics that influence its application in model evaluation. It demonstrates pronounced sensitivity to outliers due to its quadratic nature. The squaring operation causes larger errors to exert a disproportionately greater influence on the overall metric, potentially resulting in skewed evaluations when anomalous data points are present [70,71]. From a computational perspective, the MSE possesses advantageous mathematical properties, particularly its differentiability. This characteristic facilitates the implementation of optimization algorithms such as gradient descent, enabling efficient model training through analytical solutions to minimization problems [70,72]. Regarding interpretability, MSE values are invariably non-negative, with lower values indicating superior model fit to the data. However, interpretation of the MSE is somewhat complicated by its squared units, which do not directly correspond to the original measurement scale of the data [70,71].

The MAE measures the average magnitude of errors in a set of predictions, without consideration for their directionality. The mathematical formulation of MAE is given by

(2) $$\mathrm{MAE}=\frac{1}{n}\sum_{i=1}^{n}\left|y_{i}-{\hat{y}}_{i}\right|,$$

where the variables maintain the same definitions as in the MSE equation.

The MAE demonstrates distinctive statistical properties that differentiate it from the MSE. The MAE exhibits robustness to outliers, as it treats all errors with equal weight regardless of their magnitude. This characteristic proves particularly valuable when analyzing datasets containing anomalous observations that might otherwise distort the evaluation metric [70,71,72]. The MAE offers enhanced interpretability compared to the MSE, as it is expressed in the same units as the original data. For instance, an MAE value of 5 indicates that, on average, the model’s predictions deviate from actual values by five units, providing an intuitive understanding of error magnitude [70,71]. Furthermore, the MAE imposes a linear penalty structure, wherein each error contributes proportionally to the total error. This property can be advantageous in scenarios where the cost of errors remains consistent across the spectrum of predictions [70,71].

The selection between the MSE and MAE is informed by specific analytical requirements and data characteristics. The MSE is preferable in contexts where substantial errors are particularly undesirable, as its quadratic formulation imposes progressively severe penalties on larger deviations. This property makes the MSE suitable for applications where minimizing extreme errors takes precedence over average performance. Conversely, the MAE presents advantages in situations requiring straightforward interpretation of the error magnitude or when analyzing datasets potentially containing outliers that should not disproportionately influence the error metric. The linear nature of the MAE ensures that all errors contribute equally to the final metric, regardless of their magnitude.

### 3.3. Comparison Results

**Figure 2:**
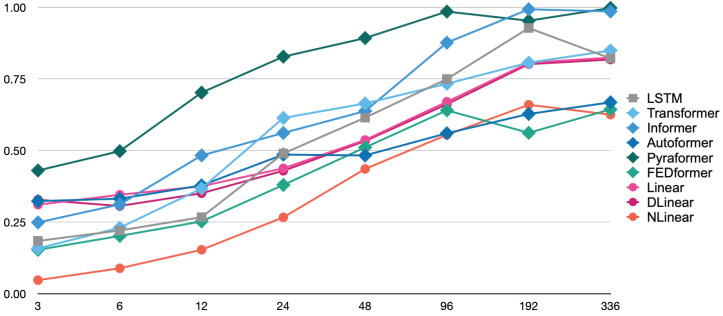
MSE trend with varying LB window size.

**Figure 3:**
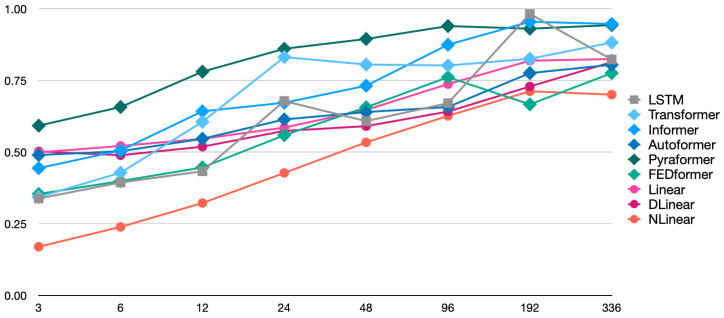
MAE trend with varying LB window size.

Figure 2 and Figure 3 illustrate the trends in the MSE and MAE as the Look-Back (LB) Window size was varied across eight values: 3, 6, 12, 24, 48, 96, 192, and 336. As shown in Figure 2, the NLinear model achieved the lowest Mean Squared Error (MSE) values for short-term to mid-term forecasts across LB windows ranging from 3 to 48 steps, highlighting its superior performance in these time horizons. Notably, its value of 0.095 at the LB Window 3 is approximately one-third of FEDformer’s 0.307, indicating remarkable accuracy. For long-term forecasting with LB windows exceeding 96 steps, the performance gap between models narrowed, though NLinear continued to exhibit relatively strong performance. While FEDformer also showed excellent results for short-term to mid-term forecasts across LB windows ranging from 3 to 24 steps, its performance declined significantly for longer horizons. In contrast, the Pyraformer model consistently exhibited the highest MSE values across all LB windows, suggesting limited suitability for the prediction task under this experimental configuration.

The MAE trend, based on the figures shown in Figure 3, also reveals that the NLinear model maintained the lowest error across all LB window sizes, demonstrating consistent superiority. Notably, there is a slight variation in the relative rankings of models when evaluated using the MSE and MAE. This discrepancy reflects differences in how each model responds to outliers or extreme prediction errors. For instance, the DLinear model showed relatively better performance in mid-term forecasts across LB windows ranging from 24 to 96 steps when evaluated using the MAE, indicating an improvement in its ranking compared to assessments based on the MSE.

In most models, an increasing trend in prediction error was observed as the LB window size expanded. This reflects the growing complexity of forecasting tasks as the temporal distance increases. However, the strength of this trend varied across models: Transformer-based architectures exhibited significant performance degradation with larger LB windows, while linear models in terms of DLinear and NLinear showed relatively mild declines.

Although the Autoformer model showed distinct behavior when switching from a 24-step to a 48-step LB window, resulting in a slightly lower MSE, this observation suggests that the self-correlation-based architecture may be effective at certain time scales, highlighting its potential for long-term forecasting.

From the experimental results, it is clear that complex transformer-based architectures do not necessarily achieve the best performance for time-series prediction tasks. Notably, the relatively simple linear model NLinear demonstrated overall superior performance across all evaluation metrics. This outcome suggests that well-designed linear models may outperform complex neural network architectures in certain time-series forecasting scenarios. The strength of NLinear lies in its combination of data normalization and linear prediction, which effectively captures underlying time-series patterns. In contrast, the strong performance of the FEDformer model underscores the utility of frequency-domain processing via Fourier transforms for extracting meaningful features from temporal data. Specifically, FEDformer’s second-place ranking in short-term forecasting highlights the importance of frequency-domain analysis in capturing periodic or cyclical patterns in time-series data.

The performance of each model exhibited distinct behaviors depending on the prediction horizon defined by the LB window size. NLinear and FEDformer excelled in short-term forecasting but experienced a decline in relative advantage as the LB window expands. In contrast, models such as DLinear and LSTM maintained relatively stable performance for long-term forecasts. This observation underscores the importance of considering the time scale of the prediction task when selecting a model for practical applications. If short-term forecasting is the primary objective, NLinear would be an appropriate choice. However, for scenarios requiring longer-term accuracy and stability, models like DLinear or FEDformer should be considered.

When comparing evaluation results using the MSE and MAE, some models exhibited changes in ranking depending on the metric used. For example, DLinear demonstrated relatively better performance when evaluated using the MAE rather than the MSE. This discrepancy arises because the MSE is sensitive to outliers or large errors, while the MAE treats all error magnitudes equally. As a result, for datasets that are particularly susceptible to outliers, it is important to evaluate models using both the MSE and MAE and select the appropriate model based on the specific application requirements. This ensures robustness against anomalies and aligns the evaluation with the task’s sensitivity to error types.

## 4. PM Prediction from 2D Images

### 4.1. System Structure

As illustrated in Figure 1, our proposed system consists of three main components: a smartphone serving as the user interface, a graphics processing unit (GPU) responsible for pre-training and fine-tuning of deep learning models, and a single-board computer (SBC) used for verification and testing. The smartphone, equipped with a monocular camera, is designed to input both images and associated environmental data. To acquire input images for predicting PM concentration, the system assumes that a camera is integrated within the smartphone.

In this study, we employed CLIP (Contrastive Language–Image Pre-Training) [73], a multimodal model designed to process both visual and textual inputs. The model is trained using pairs of images and text, which are processed through separate encoders. CLIP possesses capabilities such as image classification based on natural language instructions and numerical predictions related to associated text or data.

The backbone of CLIP utilizes transformers for both the image encoder and text encoder. Additionally, the image encoder can incorporate convolutional backbones, enabling it to balance performance and memory usage while deploying the model on SBCs. To ensure flexibility in switching architectures, this study selected vanilla CLIP [73] as the target model for implementation.

#### 4.1.1. GPU Workload Management

In this study, we implemented a GPU server equipped with two NVIDIA RTX A6000 GPUs (Santa Clara, CA, USA). The RTX A6000 is an industrial-grade GPU based on NVIDIA’s Ampere architecture, optimized for memory-intensive tasks compared to the consumer-grade RTX series. It excels in high-performance computing applications such as 3D rendering, simulation, visual computing, and deep learning. By leveraging NVIDIA’s parallel computing toolkit, Compute Unified Device Architecture (CUDA), we achieved efficient and straightforward implementation of parallel algorithms and computations. While CUDA introduces platform-specific lock-in challenges for deep learning models, its widespread adoption in this rapidly evolving field—driven by its strong market presence—has solidified its status as a de facto standard.

The NVIDIA RTX A6000 has remained a long-selling model since its market introduction in 2010, despite recent announcements of production discontinuation. As of early 2024, it continues to offer relatively high value for performance compared to other options, making it a compelling choice for applications requiring significant computational power. This GPU features 48 GB of GDDR6 memory with a 384-bit memory interface, providing substantial bandwidth at 768 GB/s. It includes 10,752 CUDA cores, 336 Tensor Cores for accelerated AI workloads, and 84 RT Cores dedicated to ray tracing, enabling efficient parallel processing across tasks. The GPU consumes up to 300 W of power and connects to the server motherboard via PCIe Gen 4, ensuring high-speed data transfer. Its catalog performance is highlighted by a maximum single-precision floating-point operations per second (FLOPS) rating of 38.71 TFLOPS, underscoring its capability to handle demanding workloads in high-performance computing environments.

In this study, we developed a system for efficiently and automatically allocating GPU resources using SLURM (Simple Linux Utility for Resource Management) [74], an open-source workload management system. SLURM is designed to handle job scheduling and resource management, and its proven use in numerous supercomputers and computing clusters has established it as a reliable solution with high efficiency and fault tolerance. Its core functionalities include allocating resources, managing job execution and monitoring, and queue administration. In resource allocation, SLURM provides users with exclusive or non-exclusive access to compute nodes, ensuring that the necessary resources are secured. For job execution and monitoring, it initiates jobs on allocated nodes and manages their operational status in real time. Through queue management, SLURM oversees pending jobs, preventing resource contention by prioritizing tasks in response to system demands. This combination of features makes it an ideal tool for optimizing GPU utilization in high-performance computing environments and the deployment of deep learning applications [75].

SLURM has also found application in single-board computers (SBCs) such as the Raspberry Pi, where its use is gaining traction as a cost-effective solution for high-performance computing environments. Yoo et al. [74] developed a high-performance cluster consisting of 68 quad-core ARMv8 64-bit Raspberry Pi 3s (Cambridge, UK). This cluster includes one master node, 64 worker nodes, a monitor node, and two storage nodes, with SLURM used for resource management and scheduling. By connecting multiple SBCs via a network to form a distributed cluster, parallel processing can be enabled, allowing for efficient management of computational resources. In particular, SLURM proves useful in scheduling long-running jobs, enabling pre-assignment of resources and streamlined job management across compute nodes.

In the current era, GPU power consumption [76] has become a critical environmental and economic challenge [77], driven by the increasing complexity of deep learning models that rely on numerous GPUs to enhance performance [78]. Given the energy efficiency of SBCs, this study has developed a platform that optimizes power consumption while maintaining adequate computational capacity for practical applications. The system is designed to enable future scalability and expandability in SBC-based environments, addressing the challenges of power limitations and computational demands.

#### 4.1.2. SBC Deployment

In deep learning applications, such as pre-training, transfer learning, and domain- or application-specific fine-tuning, GPU usage is indispensable due to the high computational demands of these tasks. However, during stages like classification or prediction where parameter updates are not required, models can also be executed on CPU-based personal computers or SBCs. In particular, SBCs serve as compact computing devices that integrate critical components of a computer system onto a single board, making them highly suitable for deploying deep learning models in resource-constrained environments.

By deploying such models on SBCs, edge computing becomes more feasible and economically viable because computational power is brought closer to the data source, reducing latency and enabling local processing. Furthermore, running deep learning models directly on SBCs eliminates reliance on remote processing units like GPUs or centralized cloud infrastructure, allowing for immediate inference processing. This capability supports real-time operations with reduced latency, significantly enhancing system independence from external resources. In particular, edge computing systems benefit from the ability to perform tasks without relying on network connectivity, thereby improving overall self-sufficiency and operational reliability.

In addition to being compact, affordable, low-power-consuming, and durable for outdoor use, SBCs have emerged as a representative solution in edge computing [79]. Their versatility and broad applicability across diverse industries have driven their increasing adoption, with a growing variety of products now available on the market [80]. Notable examples include Raspberry Pi, Banana Pi, Orange Pi, LatteePanda, BeagleBone, Asus Tinker, ODROID, and Nvidia Jetson. In this study, we selected the Raspberry Pi 5B as the SBC for implementation due to its widespread adoption and popularity in the edge computing community.

The Raspberry Pi 5B is equipped with the Broadcom BCM2712 processor, which features a 4-core ARM Cortex-A76 CPU operating at 2.4 GHz. The adoption of a 64-bit architecture significantly enhances performance compared to its predecessor, the Raspberry Pi 4B. For memory, the system utilizes LPDDR4X-4267 RAM with a maximum capacity of 8 GB, enabling faster data processing through its high-speed interface. In terms of graphics capabilities, the VideoCore VII GPU supports OpenGL ES 3.1 and Vulkan, allowing for advanced graphical rendering and high-quality video output. It also provides support for 4 K/60 Hz display resolution and dual-display configurations, making it well suited for complex visual tasks and demanding video processing applications.

The Raspberry Pi 5B features a range of input/output interfaces, including two USB 3.0 ports, two USB 2.0 ports, an HDMI 2.0 port supporting dual-display configurations, a 2.5 Gbps Ethernet port, one PCIe 2.0 interface, a 40-pin GPIO header, a camera interface with two lanes, and a display interface with four lanes. For the operating system, Raspberry Pi OS—a Debian-based distribution—was utilized, ensuring compatibility with a broad software ecosystem. In this study, since Ubuntu is adopted for the GPU server’s operating system, the Raspberry Pi is positioned as highly flexible during model porting due to its adaptability across different computing environments.

Similar to the GPU server, Raspberry Pi OS incorporates Python 3.10 package management tools such as pip and miniconda, which are lightweight virtual environments derived from Anaconda. For this study, the deep learning model development required additional dependencies beyond PyTorch 2.4.1 and torchvision, such as libjpeg-dev for JPEG image processing, libopenblas-dev to accelerate numerical computations via OpenBLAS, and libopenmp-dev to support OpenMP-based parallel computing. These libraries were installed through the APT package manager to ensure compatibility and efficient execution of the deep learning model on the Raspberry Pi platform. The venv module was used to make a virtual environment to run CLIP, and inside, the setup tools numpy, Cython, requests, torch, and torchvision packages were installed via pip.

### 4.2. Implementation Model

**Figure 4:**
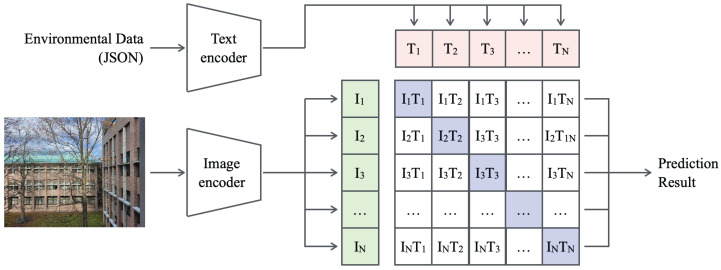
Model structure and data flow of CLIP.

In this study, we employed Contrastive Language–Image Pre-Training (CLIP) [73], a pioneering multimodal model, as the deep learning framework for estimating PM concentration from time-series images captured by a smartphone-mounted camera. Figure 4 presents the model structure and data flow of CLIP. As a multimodal model, CLIP takes text and image inputs as separate streams and processes each independently through its corresponding encoder. CLIP employs Bidirectional Encoder Representations from Transformers (BERT) [81] as its text encoder for processing textual inputs, serving as a baseline. For visual processing, the image encoder can adopt either ResNet-based or Vision Transformer (ViT)-based backbones. The feature representations generated by both encoders are structured into a relational matrix that captures interactions between text and images. This mechanism facilitates pre-training on large-scale text–image pairs, enabling CLIP to associate visual concepts with corresponding textual descriptions.

The features of CLIP [73] include not only multimodal learning [82] but also contrastive learning [83] and zero-shot learning [84]. In multimodal learning, CLIP processes both text and images simultaneously, integrating their feature representations to enable the model to understand natural language descriptions of visual content and generate images from textual inputs. In contrastive learning, CLIP utilizes a contrastive loss function to train the model by aligning semantically related image–text pairs in the embedding space while repelling dissimilar pairs. This strategy improves the model’s ability to recognize diverse visual concepts and associate them with their textual counterparts, thereby enhancing its generalization across unseen data.

Pre-training CLIP using approximately 400 million image–text pairs automatically collected from the internet has enabled it to generalize across diverse concepts and scenarios by leveraging its extensive and varied training data [73]. Zero-shot learning further enhances this generalization capability by allowing CLIP to classify or recognize new images without additional task-specific training. This approach empowers CLIP to achieve high accuracy in previously unseen tasks, relying on its pre-training on large-scale multimodal data.

In this study, PM concentration was estimated from time-series images captured by a smartphone camera. Because PM concentration affects visual characteristics such as sky color, visibility clarity, and object sharpness, CLIP identifies these specific features associated with PM density. The visual features extracted by CLIP are further used to generate textual descriptions of PM concentrations, which are combined with diverse data sources, including weather information and historical concentration distributions. By harnessing the zero-shot learning capability, the model can estimate PM concentrations in unfamiliar environments or from different cameras without requiring additional task-specific training. This approach enables robust estimation across a wide range of scenarios and imaging conditions.

### 4.3. Implementation Details

Since CLIP [73] was introduced, numerous improved variants and derivative models, such as ALIP [85], BLIP [86], DLIP [87], ELIP [88], FLIP [89], GLIP [90], MLIP [88], and X-LIP [91] have been proposed in a short period, reflecting the rapid evolution of multimodal learning frameworks. For this study, we focused on using vanilla CLIP [73] as a baseline model. Although the original CLIP implementation is publicly available on GitHub (https://github.com/openai/CLIP) under the MIT license, it offers only six backbone architectures: four ResNet-based models and two ViT-based models. To address this limitation, we adopted OpenCLIP [92], a fork of the original CLIP repository that enables a broader selection of backbone architectures. OpenCLIP provides three ResNeXT-based models in addition to extended ViT variants, including Big, Large, Huge, and Giant configurations. This expansion enables greater flexibility for customization and adaptation to a wide range of tasks and datasets. Building on recent advances in CLIP [93], we expect compatibility with diverse applications while maintaining model robustness across various use cases.

### 4.4. Evaluation Datasets

**Figure 5:**
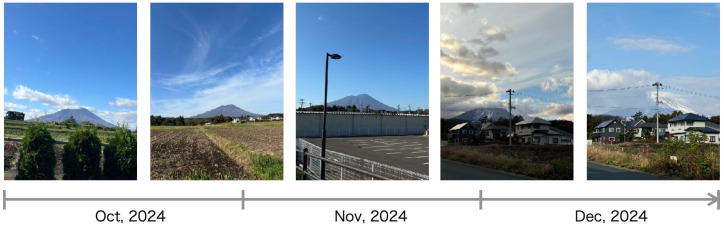
Example images of the captured data taken in Takizawa City, Iwate Prefecture, Japan.

The image data were captured using three different smartphone models. Table 2 lists the major specifications of the cameras embedded in each smartphone. Over a three-month period from October to December 2024, 30 images were collected at irregular intervals. The resolution of each image varied depending on the specific camera model used. Example images of the captured data are shown in Figure 5, all taken in Takizawa City, Iwate Prefecture, Japan, where the research team is based. In the background of these images lies Mount Iwate, a stratovolcano with an elevation of 2038 m.

Takizawa City is located at a latitude of $39^{\circ }73^{\prime }47^{\prime \prime }\;N$ and a longitude of $141^{\circ }07^{\prime }70^{\prime \prime }\;E$, as per the municipal office. Its area is approximately $182.32\;{\mathrm{km}}^{2}$. The city lies within a humid temperate climate zone and experiences four distinct seasons. Due to its inland position in the Tohoku region, this city is subject to a continental climate influence, which results in pronounced seasonal temperature variations. The annual average temperature in Takizawa is approximately 11 °C. Summer periods are generally warm, with daytime temperatures in the range of 25~30 °C and nighttime temperatures typically falling to 15~20 °C. In contrast, winter periods are cold, with daytime temperatures usually in the range of 0~5 °C and nighttime lows often dropping to −5~−10 °C. Spring and autumn are transitional seasons characterized by rapid temperature changes and significant diurnal temperature variation, especially between morning and evening.

The images from left to right in the figure represents the temporal sequence of the period. During the first half of this time, clear autumn skies were frequent, resulting in tranquil landscapes characterized by open vistas. In contrast, the latter half of the period featured prolonged cloud cover, as the scenery gradually progressed into the snow season, exhibiting a more dramatic and textured visual aesthetic.

**Figure 6:**
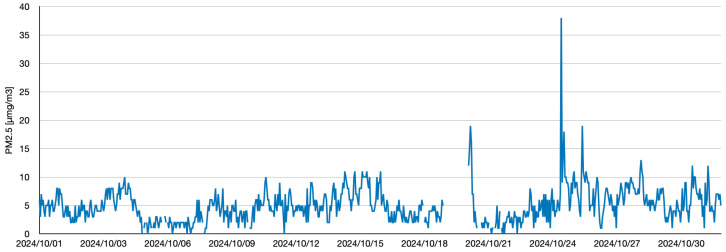
Time-series data of PM_2.5_ for one month, sampled at 1 h intervals. Notable gaps are present in several timeframes due to missing data.

**Figure 7:**
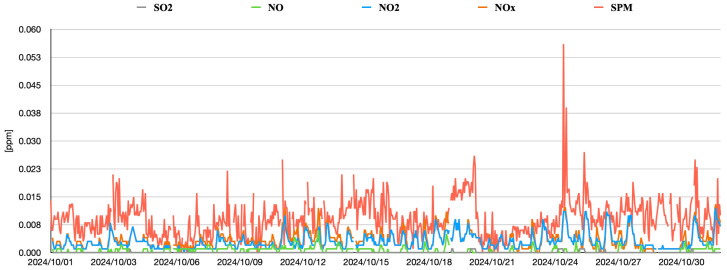
Time-series data of SO_2_, NO, NO_2_, NO_x_, and SPM for one month, sampled at 1 h intervals. Notable gaps are also present in several timeframes due to missing data.

Figure 6 and Figure 7 depict time-series changes in the data retrieved via the Soramame-kun API provided by Japan’s Ministry of the Environment. To ensure reusability and generalizability, the data were stored in JSON format. This format is especially well suited for deep learning tasks, owing to its compact structure and readable syntax. The system supports high-precision prediction, scalability, and efficiency, enabling efficient data handling and application deployment. To ensure adaptability across diverse scenarios, JSON was adopted as the default data format for practical deployment.

### 4.5. Experiment Results

We evaluated prediction accuracy using the Top-1 and Top-5 metrics. The Top-1 accuracy measures the proportion of cases where the most probable predicted class matches the actual correct label. Specifically, it counts instances where the highest probability assigned to the predicted class aligns with the true label. On the other hand, the Top-5 accuracy refers to the proportion of cases where the true label is among the top five predicted classes. While the Top-1 accuracy is essential for tasks requiring strictness, the Top-5 accuracy provides a more lenient evaluation standard, allowing the true label to be identified within the top five probability predictions. This distinction ensures that evaluation criteria align with the specific requirements of the task at hand. For example, the Top-1 accuracy is suitable for tasks where a single correct answer is expected, whereas the Top-5 accuracy is appropriate when the true label must be selected from multiple possible options.

As shown in Table 3, an example result from the proposed model for the time slot 09:00–10:00 on 5 October 2024 is presented. The model outputs are sorted by descending probability. In this case, the highest-probability prediction matches the Ground-Truth (GT) value (2 μg/m^3^) and is classified as Top-1. The model was applied to all images in the dataset, yielding a Top-1 accuracy of 0.24 and a Top-5 accuracy of 0.52 across 50 images. Since PM concentration is influenced by numerous factors, as shown in Figure 7, this study opted to evaluate predictions using the Top-5 accuracy rather than standard classification metrics, focusing only on probability values near the true values. Therefore, in this experiment, the Top-5 accuracy was defined as a ±2 μg/m^3^ range around the true value to account for the inherent challenges of predicting PM concentrations at 1 μg/m^3^ resolution. This approach ensured that the Top-5 metric reflected realistic prediction capabilities while avoiding overly restrictive evaluation criteria.

After confirming that CLIP functioned correctly on our GPU-based SLURM system, we deployed it on an SBC (Raspberry Pi 5B 8GB) to evaluate processing performance. The experimental results showed that for 50 images, the total inference time was 14.86 s on the GPU and 133.82 s on the SBC, indicating a speed difference of approximately nine times, which means the GPU is up to nine times faster than the SBC. Given the trade-offs between power consumption and cost, deploying the system on an SBC remains a practical choice. For individual image processing, the GPU delivered inference times of 0.29 s, while the SBC required 2.68 s, highlighting the significant performance gap in favor of the GPU. Even with frequent changes in PM concentrations over time, the 2.68-s delay on the SBC provides sufficient time for accurate predictions. This balance of energy efficiency, affordability, and acceptable latency ensures that our proposed system is viable for deployment, even when considering the slower processing speed of the SBC.

## 5. Discussion

This study introduces a novel framework for predicting PM concentrations using a multimodal deep learning approach that incorporates both time-series data collected via an AQ monitoring device [45] and visual information captured by smartphone cameras. The integration of CLIP into environmental monitoring tasks represents a significant advancement, as it enables the model to associate visual cues with PM levels through contrastive learning. We consider that this approach addresses the limitations of traditional sensor-based systems by offering a scalable and cost-effective alternative for real-time AQ assessment. The comparative analysis of time-series forecasting models revealed that simpler linear models, particularly NLinear, outperformed more complex transformer-based architectures [8] in short-term prediction tasks. This finding aligns with recent studies suggesting that linear models can be more robust and efficient for specific forecasting horizons.

The superior performance of NLinear [56] in both the MSE and MAE metrics underscores the importance of model selection based on task-specific requirements rather than architectural complexity alone. In contrast, transformer-based models demonstrated advantages in capturing long-term dependencies and periodic patterns, particularly when using frequency-domain techniques like those in FEDformer [62] or Informer [60]. However, their computational demands and sensitivity to hyperparameter tuning pose challenges for deployment in resource-constrained environments. These trade-offs highlight the need for hybrid models that can balance accuracy and efficiency, especially for applications requiring both short- and long-term forecasting capabilities. The image-based prediction component, powered by CLIP, achieved a Top-1 accuracy of 24% and a Top-5 accuracy of 52%, demonstrating the feasibility of estimating PM concentrations from visual data. The ability of CLIP to generalize across diverse scenes and lighting conditions without task-specific fine-tuning is particularly valuable for real-world deployment, where environmental variability is high.

The system architecture, which combines GPU-based training with SBC-based inference, offers a practical solution for edge deployment. Despite its limited computational power, the Raspberry Pi 5B was able to perform inference within 2.68 s per image, which is sufficient for near-real-time applications. This dual-platform strategy not only reduces reliance on cloud infrastructure but also supports decentralized monitoring, which is crucial for large-scale environmental sensing in remote or underserved areas. Nevertheless, the dataset used for image-based prediction was collected over a three-month period, limiting its ability to capture seasonal variations in PM levels. Since air quality is influenced by factors such as temperature, humidity, and wind patterns, extending the dataset to cover a full year would enhance the robustness and generalizability of our proposed model. Additionally, the relatively small number of images may limit the statistical significance of the results, highlighting the need for larger and more diverse image datasets in future research.

We consider that another aspect of improvement lies in the model architecture. While CLIP’s zero-shot learning capabilities are impressive, incorporating domain-specific fine-tuning or integrating additional modalities, such as meteorological data, satellite imagery, or sensor readings, could further improve prediction accuracy. Recent models like AQP-Mamba [52], which combine video data with structured state space modeling, demonstrate the potential of multimodal fusion for enhancing environmental monitoring systems. Therefore, this study demonstrates the viability of using multimodal deep learning [94] for PM prediction, bridging the gap between high-performance computing and low-power edge deployment. The findings suggest that further refinement such as architectural optimization, dataset expansion, and multimodal integration could lead to significant improvements. Our approach could thus serve as a foundation for scalable, real-time air quality monitoring systems. Future research should explore these directions to fully realize the potential of AI-driven environmental sensing.

## 6. Conclusions

This study aimed to develop a framework for predicting PM concentrations using mobile cameras integrated into smartphones. The proposed approach employs a transformer-based multimodal deep learning model, which utilizes CLIP as a pioneering multimodal system that processes image–text pairs by compressing visual features into tokens and encoding semantic relationships via 2D matrices. The performance of the model was evaluated on custom datasets tailored for environmental monitoring tasks. To compare processing efficiency, our system was implemented on both GPU and SBC platforms. While there was a significant performance gap between the GPU and SBC systems, this study emphasized the practicality of deploying SBCs due to their low power consumption and cost-effectiveness. Despite limitations in computational capacity, the framework enables real-time prediction during idle periods, even under frequent fluctuations in PM concentrations. By integrating GPU-based training with SBC-powered edge deployment, this feasibility study demonstrates a viable pathway for practical implementation, bridging high-performance computing with energy-efficient hardware to address environmental monitoring challenges.

As future challenges, we aim to improve performance by exploring alternatives to the transformer-based backbone in CLIP, including replacing it with a large multilayer perceptron or implementing hybrid architectures that combine both transformer and multilayer perceptron components. Additionally, while this study utilized a dataset spanning three months, extending the evaluation to datasets covering over one year would enable more comprehensive analysis of seasonal variations in PM concentration dynamics. By incorporating long-term datasets, we aim to gain deeper insights into how environmental factors influence prediction accuracy across different seasons, thereby enhancing the model generalizability for real-world deployment.

## Figures and Tables

**Table 1:** Summary of PM_2.5_ forecasting studies.

Authors	Methodology	Data Source Location
Koo et al. [46]	ConvLSTM-DNN	Seoul, South Korea
Feng et al. [47]	LSTM and RF	Beijing and Tianjin, China
Jianyao et al. [48]	ConvLSTM-DNN	Seoul, South Korea
Zhang et al. [49]	PMEstimatingNet	China and the USA
Dai et al. [50]	LUR-GBM	China
Mohan et al. [51]	En3C-AQI-Net	Delhi, India
Ahmed et al. [52]	AQE-Net	Karachi, Pakistan
Ahmed et al. [53]	AQP-Mamba	Karachi, Pakistan

**Table 2:** Major specifications of cameras embedded in smartphones.

Model Name	SH-RM15	SCG24	iPhone 15
image resolution [pixel]	4000 × 2250	4000 × 3000	4000 × 3000
number of images	10	10	30

**Table 3:** Experimental results for sample data collected during time slot 09:00–10:00 on 5 October 2024. The GT value is 2 μg/m^3^, obtained from Soramame-kun.

Top-	Predicting PM_2.5_ Value (μg/m^3^)	CLIP Output Probability
**1**	2	0.1527
2	1	0.1391
3	3	0.1066
4	6	0.0941
**5**	7	0.0941
-	9	0.0912
-	8	0.0830
-	10	0.0780
-	4	0.0733
-	5	0.0481
-	0	0.0398

## Data Availability

The raw data supporting the conclusions of this article will be made available by the authors upon request.

## Abbreviations

The following abbreviations are used in this manuscript:

API	Application Programming Interface
BERT	Bidirectional Encoder Representations from Transformers
CLIP	Contrastive Language–Image Pre-Training
CMAQ	Community Multiscale Air Quality
CNN	Convolutional Neural Network
CUDA	Compute Unified Device Architecture
DNN	Deep Neural Network
FLOPS	Floating-Point Operations Per Second
GPU	Graphics Processing Unit
GRU	Gated Recurrent Unit
GT	Ground Truth
JSON	JavaScript Object Notation
LB	Look-Back
LSTM	Long Short-Term Memory
MAE	Mean Absolute Error
MSE	Mean Squared Error
PM	Particulate Matter
RNN	Recurrent Neural Network
SBC	Single-Board Computer
SSSM	Structured Selective State Space Model
SLURM	Simple Linux Utility for Resource Management
ViT	Vision Transformer
WRF	Weather Research and Forecasting
